# *B-MOBILE* - A Smartphone-Based Intervention to Reduce Sedentary Time in Overweight/Obese Individuals: A Within-Subjects Experimental Trial

**DOI:** 10.1371/journal.pone.0100821

**Published:** 2014-06-25

**Authors:** Dale S. Bond, J. Graham Thomas, Hollie A. Raynor, Jon Moon, Jared Sieling, Jennifer Trautvetter, Tiffany Leblond, Rena R. Wing

**Affiliations:** 1 Department of Psychiatry and Human Behavior, Alpert Medical School of Brown University, The Miriam Hospital/Weight Control and Diabetes Research Center, Providence, Rhode Island, United States of America; 2 Department of Nutrition, University of Tennessee, Knoxville, Tennessee, United States of America; 3 MEI Research Ltd, Edina, Minnesota, United States of America; King's College London, United Kingdom

## Abstract

**Purpose:**

Excessive sedentary time (SED) has been linked to obesity and other adverse health outcomes. However, few sedentary-reducing interventions exist and none have utilized smartphones to automate behavioral strategies to decrease SED. We tested a smartphone-based intervention to monitor and decrease SED in overweight/obese individuals, and compared 3 approaches to prompting physical activity (PA) breaks and delivering feedback on SED.

**Design and Methods:**

Participants [N = 30; Age  = 47.5(13.5) years; 83% female; Body Mass Index (BMI) = 36.2(7.5) kg/m^2^] wore the SenseWear Mini Armband (SWA) to objectively measure SED for 7 days at baseline. Participants were then presented with 3 smartphone-based PA break conditions in counterbalanced order: (1) 3-min break after 30 SED min; (2) 6-min break after 60 SED min; and (3) 12-min break after 120 SED min. Participants followed each condition for 7 days and wore the SWA throughout.

**Results:**

All PA break conditions yielded significant decreases in SED and increases in light (LPA) and moderate-to-vigorous PA (MVPA) (*p*<0.005). Average % SED at baseline (72.2%) decreased by 5.9%, 5.6%, and 3.3% [i.e. by mean (95% CI) −47.2(−66.3, −28.2), −44.5(−65.2, −23.8), and −26.2(−40.7, −11.6) min/d] in the 3-, 6-, and 12-min conditions, respectively. Conversely, % LPA increased from 22.8% to 26.7%, 26.7%, and 24.7% [i.e. by 31.0(15.8, 46.2), 31.0(13.6, 48.4), and 15.3(3.9, 26.8) min/d], and % MVPA increased from 5.0% to 7.0%, 6.7%, and 6.3% (i.e. by 16.2(8.5, 24.0), 13.5(6.3, 20.6), and 10.8(4.2, 17.5) min/d] in the 3-, 6-, and 12-min conditions, respectively. Planned pairwise comparisons revealed the 3-min condition was superior to the 12-min condition in decreasing SED and increasing LPA (*p*<0.05).

**Conclusion:**

The smartphone-based intervention significantly reduced SED. Prompting frequent short activity breaks may be the most effective way to decrease SED and increase PA in overweight/obese individuals. Future investigations should determine whether these SED reductions can be maintained long-term.

**Trial Registration:**

ClinicalTrials.gov NCT01688804

## Background

Excessive time spent in sedentary behaviors, activities that require very low energy expenditure and occur while sitting or lying down [1]–[2], has become a prominent health concern. Evidence continues to accumulate suggesting that greater total time spent in sedentary behavior increases the risk for obesity [3]–[4], poor cardiometabolic health [5]–[7] and mortality [8]–[9], independent of physical activity level. Thus, the deleterious health impact of sedentary behavior may not be completely mitigated by habitual physical activity.

More refined analysis of sedentary behavior demonstrates that not only is the total volume of sedentary behavior important, but also the pattern in which it is accumulated. Observational studies suggest that regular interruption of sedentary behavior with brief physical activity breaks of at least a light intensity is associated with a more favorable cardiometabolic risk profile compared to accumulating sedentary behavior in longer, uninterrupted bouts [6], [10]. Recent laboratory experiments have provided causal evidence that breaking up sitting time with short bouts of light- or moderate-intensity walking has beneficial acute effects on postprandial glucose and insulin responses [11]–[13], energy expenditure [14], and expression of skeletal muscle genes involved in adaptive cellular processes and carbohydrate metabolism [15].

Despite the potential to minimize health risks associated with prolonged bouts of sedentary behavior via habitual interruption with brief physical activity breaks, few interventions have been conducted to promote adoption of this relatively small behavior change. Moreover, many of the different intervention approaches that have been employed, including an electronic television lock-out system [16], a portable pedal machine, pedometer, and accompanying motivational website [17], and a single individual goal-setting session plus written materials [18], have produced only modest reductions (3.2%–3.8%) in objectively-measured sedentary time. Other interventions have produced larger reductions in sedentary time, although they were either limited to the workplace setting and involved use of expensive equipment solely to reduce sedentary behavior [19] or required more intensive lifestyle approaches involving laborious self-monitoring of sedentary behavior [20]. Thus, there is a clear need for low-intensity interventions that produce substantial reductions in sedentary behavior across multiple environmental domains.

Sitting, unlike exercising, is highly habitual, can occur many times throughout the day totaling many hours, and often involves little or no conscious processing or planning. Therefore, interventions to break up and reduce sedentary time should be simple, require minimal forethought or effort, with the ability to be implemented easily in most environments, and should automatically elicit a reaction of standing up (and ideally walking) upon exposure [21]. A mobile health (mHealth) intervention has the potential to fulfill all of the above criteria via smartphone technology. Smartphones are owned by 61% of individuals in the U.S. (with the highest rates of ownership among ethnic/racial minority groups), who use the devices for over 2 hours per day on average [22]–[23]. Smartphones represent a unique opportunity for intervening on sedentary behavior given that they can automatically monitor time spent in sedentary behavior via an onboard accelerometer, thereby eliminating the need for laborious self-monitoring. These data can then be used to deliver individually-tailored behavioral prompts and reinforcing feedback in real-time in the natural environment to interrupt prolonged periods of sedentary behavior with brief physical activity breaks. It is also possible to present feedback in an entertaining and engaging format using game elements that promote adherence to the intervention protocol. While smartphones have been used in the past to encourage increases in physical activity [24], we believe this is the first attempt to use smartphones to automatically monitor and prompt reductions in sedentary behavior.

The overall aim of this trial was to test a smartphone-based intervention designed to decrease objectively-measured sedentary time in overweight/obese individuals by breaking up prolonged periods of sedentary behavior with brief physical activity (i.e. walking) breaks. Given that the most effective strategy for maximizing break frequency and duration is unknown, this study also compared 3 different conditions, each followed for a 7-day period and presented in a counterbalanced order, to prompting physical activity breaks and delivering feedback on time spent in sedentary behavior: (1) 3-min break prompt after 30 continuous sedentary minutes; (2) 6-min break prompt after 60 continuous sedentary minutes; and (3) 12-min break prompt after 120 continuous sedentary minutes. The 3 conditions were also compared on time spent in light- and moderate-to-vigorous intensity physical activity. We hypothesized that: 1) all 3 conditions would produce both significant reductions in time spent sedentary and increases in light- and moderate-to-vigorous physical activity compared to baseline; and 2) one or more of the activity break conditions would produce superior improvements in time spent sedentary and time spent performing light and moderate-to-vigorous physical activity compared to the other condition(s).

## Methods

### Ethics Statement

This project was approved by the institutional ethics review board at The Miriam Hospital, Providence, Rhode Island, USA. Written informed consent was obtained for all participants, in accordance with the principles expressed in the Declaration of Helsinki.

The protocol for this trial and supporting TREND checklist are available as supporting information; see Checklist S1 and Protocol S1.

### Subjects, Recruitment and Determination of Eligibility

A convenience sample of men and women were recruited from August 2012 through November 2013 via *B-MOBILE* study advertisements placed in local newspapers and on research hospital network-affiliated intranet/internet sites and social media outlets (i.e. Facebook and Twitter). Persons interested in participating were asked to contact the research center by calling a provided telephone number or visiting a website.

**Figure 1:**
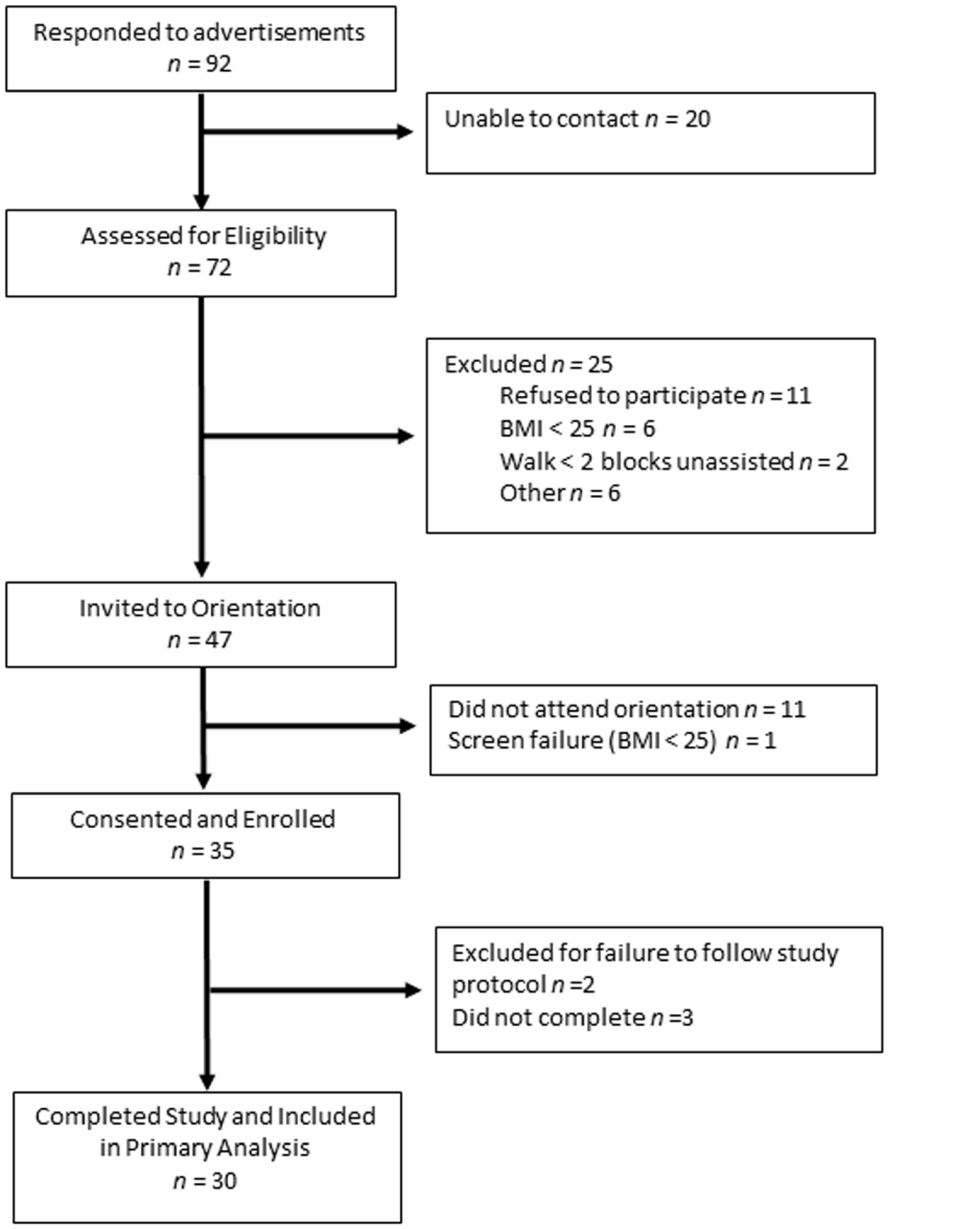
Flow diagram includes data on number of respondents to study advertisements, participant enrollment, number of participants who completed the study, and primary analysis.

Individuals were screened by phone to determine study eligibility. Participants were 21 to 70 years of age, and overweight or obese [Body Mass Index (BMI)≥25 kg/m^2^] given that overweight/obesity is a risk factor for engagement in high levels of sedentary behavior [3]–[4]. Individuals were not excluded based on physical activity level given that sedentary behavior poses morbidity and mortality risk independent of physical activity [5]–[9]. As shown in Figure 1, 72 individuals responded to advertisements and underwent telephone screening. Of the 47 who met inclusion criteria and were invited to a study orientation visit, 35 attended and enrolled. Of these 35, 2 were excluded for failure to follow the study protocol, 3 dropped out of the study, and 30 completed the study and were included in analyses. All 30 participants who completed the study did so between September 2012 and December 2013.

### Study Design and Procedures

The *B-MOBILE* study employed a within-subjects design to examine the short-term efficacy of a smartphone-based intervention to reduce sedentary time in overweight/obese individuals and to determine which of three physical activity break conditions yielded the greatest reduction in sedentary time. Participants reported to the research center on 5 separate occasions over a 4-week period. At the first visit, participants provided informed consent, completed a demographics questionnaire, underwent height and weight measurements, and were given a multi-sensor armband monitor to wear and objectively measure time spent in sedentary behavior for 7 consecutive days. This initial week long period provided a baseline assessment for all subsequent comparisons.

After the 7-day baseline period, participants returned for a second study visit during which they first received 10 minutes of in-person education from a trained research staff member to provide a rationale for reducing sedentary behavior. Topics covered included defining sedentary behavior, risks of excessive time spent in sedentary behavior, and potential benefits to modifying time spent in sedentary behavior. Next, participants were provided with an Android smartphone (i.e. *Samsung Exhibit* 4G SGH-T759) configured with the *B-MOBILE* application (aka. app) to reduce time spent in sedentary behavior (see detailed description of the *B-MOBILE* app and features in *Intervention* section below). Participants received a brief tutorial on the features of the smartphone and *B-MOBILE* app and the first of 3 smartphone-based physical activity break conditions (i.e. 3-min break after 30 continuous sedentary minutes, 6-min break after 60 continuous sedentary minutes, or 12-min break after 120 continuous sedentary minutes) in counterbalanced order. Participants followed each protocol for 7 consecutive days and continued to wear the armband monitor simultaneously as an objective measure of time spent in sedentary behavior. After completion of each condition, participants returned to the research center to be informed of the next condition to which they were randomized and to recharge the armband monitor for continued use. At the final visit, participants completed a survey designed to assess acceptability of the *B-MOBILE* app overall and physical activity break conditions, and received printouts from the armband monitor displaying time spent in sedentary behavior and different intensities of physical activity. Participants received a $20 honorarium at the conclusion of each of the five visits for a total compensation of $100. This study was approved by The Miriam Hospital Institutional Review Board, Providence, Rhode Island, USA.

### B-MOBILE Smartphone-Based Intervention

The intervention approach combined a smartphone device with an onboard accelerometer and a smartphone app designed in collaboration with behavioral (DSB, JGT) and engineering/computer (JM, JS) scientists. The intervention components are detailed below.

#### Real-time monitoring of Sedentary Behavior

The purpose of this component was to automatically monitor participants' sedentary behavior in real time via the smartphone's onboard accelerometer. Monitoring of behavior is a key component of behavior change interventions but can increase participant burden when traditional paper-and-pencil diaries are used [25]–[26]. This concern is particularly relevant with sedentary behavior which is highly habitual and occurs frequently and in different forms throughout the day [21].

Participants were instructed to carry the smartphone on their person (in a pocket or secured to clothing via a clip) at all times during the intervention period. The smartphone's accelerometer was programmed to monitor sedentary behavior [≤1.5 metabolic equivalents (METs)] in 1-minute increments. Accelerometry data were converted to metabolic units using an algorithm adapted from Fujiki and colleagues [27]. The smartphone and armband monitor were significantly correlated in percentage of daily time that current study participants spent sedentary across the 3-week intervention period (Pearson's *r* = 0.53, 95% CI: 0.21, 0.75, *p* = 0.003). Monitored sedentary data were available to the research team in real-time via the smartphones' always-on Internet connection and were used to inform the subsequently described automated goal-setting, prompting, and feedback intervention components.

#### Sedentary behavior goal-setting, prompting, and feedback

**Figure 2:**
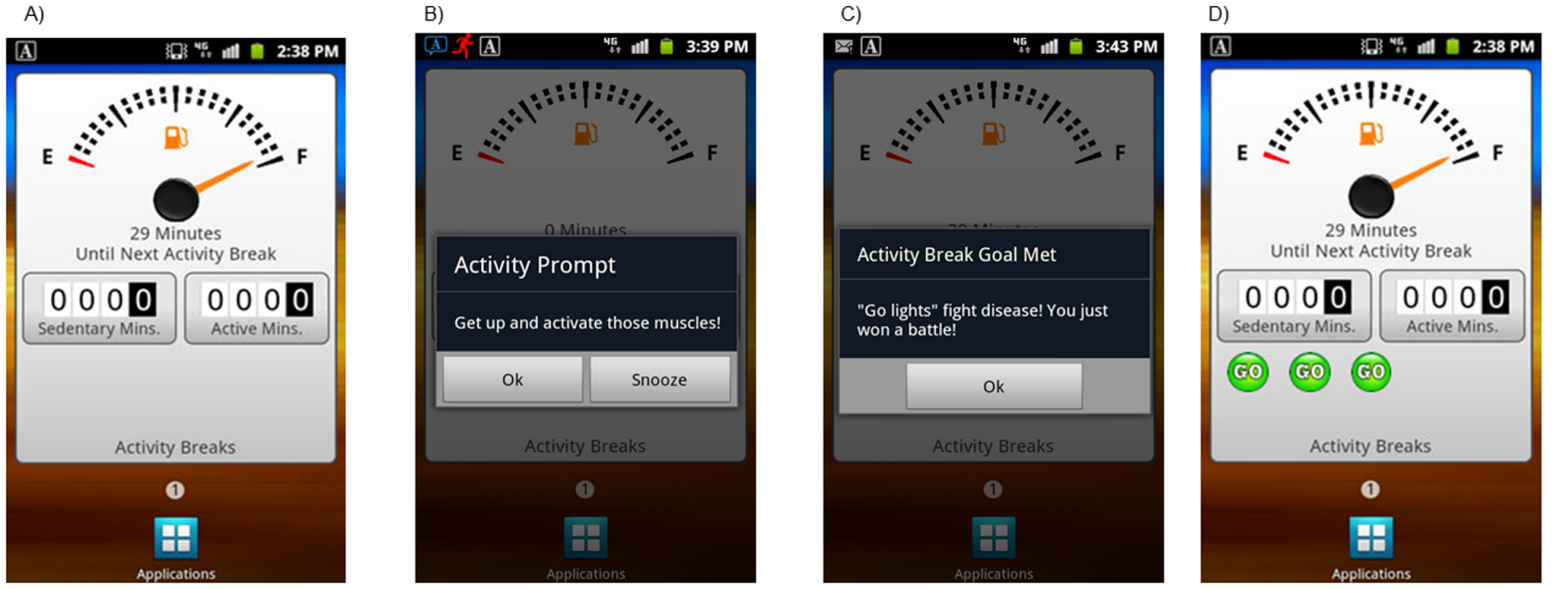
Smartphone display when A) smartphone is activated and idle, B) an activity prompt is presented, C) the onboard accelerometer detects that the activity break goal has been accomplished, D) “Go lights” have been earned by performing activity breaks following activity prompts.

These components of the intervention were presented within the context of an automobile dashboard metaphor that was visible when the smartphone display was active. As shown in Figure 2, the primary features of the dashboard included: 1) a “fuel gauge” depicting the number of sedentary minutes remaining until the next physical activity break; and 2) two odometers tracking the total number of sedentary minutes and activity minutes (of any intensity) accumulated that day.

The three physical activity break goals [i.e. (1) 3-min walking break after 30 continuous sedentary minutes, (2) 6-min walking break after 60 continuous sedentary minutes, and (3) 12-min walking break after 120 continuous sedentary minutes] were used in conjunction with the real-time accelerometry data from the smartphone to prompt physical activity breaks when the maximum number of continuous minutes spent in sedentary behavior was reached. Using the first goal as an example, when the smartphone accelerometer detected that a participant had accrued 30 minutes of time spent in sedentary behavior without taking a physical activity break of at least 3 minutes, the smartphone produced an audible prompt with an on-screen text reminder of the physical activity break goal and encouragement for performing a physical activity break. Participants had the option of responding to the prompt by performing a physical activity break, silencing the prompt, or delaying the prompt to reoccur after a set period of 30 minutes.

Real-time accelerometry data from the smartphone were used to determine whether participants were compliant with the prompts to take a physical activity break. When a participant successfully responded to a prompt by performing a physical activity break for the recommended duration, they received a message praising their accomplishment. Additionally, a bright green “go” light appeared on the dashboard each time participants fully complied with the physical activity break prompt, up to a total of 10 “go” lights per day. The persistent display on the smartphone screen was also updated to indicate that the fuel gauge had been “refilled.” Persistent displays such as these have been shown to motivate health behavior in other studies using similar technology [24], [28]–[29].

### Objective Assessment of Sedentary Behavior and Physical Activity Outcomes

The SenseWear Mini Armband monitor (SWA; BodyMedia, Inc., Pittsburgh, PA) was used to objectively measure time spent in sedentary behavior (primary outcome) and physical activity of both a light and moderate-to-vigorous intensity (secondary outcomes) during baseline and each of the 3 physical activity break conditions. The SWA is a wireless multi-sensor monitor worn over the upper right triceps muscle that simultaneously integrates movement data from a triaxial accelerometer, physiologic metrics from sensors measuring heat flux, galvanic skin response, skin and near-body temperatures, and sex, age, body weight, and height to estimate energy expenditure and intensity of activities using proprietary software algorithms (SenseWear Professional Software, version 7.0). The SWA has been shown to accurately measure daily energy expenditure against criterion measures [30]–[31] and provide estimates of time spent in sedentary behavior and physical activity comparable to other objective monitors [32]–[33]. Additionally, the SWA has been increasingly used to quantify sedentary time in overweight/obese individuals [34]–[35].

Subjects were asked to wear the SWA during all waking hours for 28 consecutive days across the 7-day baseline and 21-day intervention periods. Time spent in sedentary behavior and physical activity of a light and moderate-to-vigorous intensity was determined using MET values. Activities with MET values of ≤1.5, >1.5 and <3, and ≥3 were considered as sedentary behavior, light physical activity, and moderate-to-vigorous physical activity, respectively. For data to be considered valid, participants needed to have worn the SWA for ≥8 h/d on ≥4 d (including ≥1 weekend day) during the 7-day baseline period and each of the three 7-day physical activity break conditions. All participants met the criteria, wearing the SWA for an average of 13.0±1.5 h/d on 6.7±1.1 d across the total 28-day study period.

### Statistical Analysis

The primary aim of the study was to determine the magnitude of reduction in time spent in sedentary behavior produced by each of the 3 physical activity break conditions, to compare time spent sedentary at baseline versus during each of the 3 activity break conditions, and to determine whether the reductions in sedentary behavior produced by any of the 3 conditions was superior to the others. An *a priori* power analysis determined that a sample size of at least N = 20 was necessary to detect a difference of ≥5% in time spent in sedentary behavior for a series of planned paired pairwise comparisons testing baseline versus each activity break condition and each activity break condition versus the others, conducted in the context of a repeated measures analysis of variance. Assumptions of the power calculation included a pooled standard deviation of ≤10% in time spent sedentary across all 4 weeks, two-sided tests, and alpha  = .05.

Despite wear time requirements for the devices used to objectively monitor sedentary behavior, studies of sedentary behavior that employ a within-subjects design are complicated by the possibility of differences in monitor wear time between the within-subject conditions (i.e., baseline and the 3 physical activity break conditions). Therefore, the primary analysis investigating changes in time spent in sedentary behavior, which is described in greater detail below, was performed with the outcome coded as percent of total time spent in sedentary behavior within each condition. In order to represent changes in sedentary behavior in the metric of minutes, a secondary analysis was conducted in which the difference in sedentary minutes between baseline and each of the physical activity break conditions was estimated using the following formula: (% daily time spent sedentary during baseline week - % daily time spent sedentary during condition X week) x (average min/d of SWA monitor wear time across baseline and condition X weeks)/100 – e.g., (74.6% time spent sedentary at baseline −67.2% time spent sedentary at condition X week) x (856.3 min/d average SWA monitor wear time across baseline and condition X weeks)/100 = 63.4 min/d reduction in sedentary time between baseline and condition X. The same two-step approach was also applied to analyses of light- and moderate-to-vigorous intensity physical activity.

Descriptive statistics were calculated for subject characteristics and baseline levels of the outcome measures. Repeated measures analysis of variance was used to compare time spent in sedentary behavior at baseline and in the 3 physical activity break conditions. Planned pairwise comparisons were conducted to compare the 3 physical activity break conditions to the baseline week and each other. Cohen's d effect size was computed for the change from baseline after each of the 3 physical activity break conditions. The same procedures were used to evaluate changes in secondary outcomes related to time spent in physical activity. These outcomes analyses were repeated controlling for the order in which the physical activity break conditions were administered. These results are not reported as there was no effect of order of presentation on the pattern of results. All analyses were performed using SPSS Statistics for Windows (version 20.0; SPSS, IBM Corp, Armonk, NY). All tests of statistical significance were two-tailed, with α = 0.05.

## Results

### Subject Characteristics

Subject characteristics are presented in Table 1. On average, subjects were middle-aged and obese. The majority of subjects was female, white, non-Hispanic, and employed with at least some years of college education. Of the 12 participants who reported not being employed, 4 were homemakers, 4 were retired, 3 were currently looking for employment, and 1 was a student.

**Table 1:** Subject characteristics (N = 30).

Age (Mean ± SD years)	47.5±13.5
% Female	83.3
% Race	
White	66.7
African-American	13.3
American Indian/Alaskan Native	3.3
Asian	3.3
Other	13.3
% Hispanic	10.0
% Marital status	
Never married	40.0
Married	43.3
Divorced	16.7
% Education level	
No college	20.0
Some college	40.0
College graduate	40.0
% Full or part-time employed	60.0
Professional, administrator or executive	52.9
Clerical work, administrative support, sales, or technician	47.1
BMI (Mean ± SD kg/m^2^)	36.2±7.5
Weight (Mean ± SD kg)	98.1±21.6

Note. BMI =  Body Mass Index (kg/m^2^).

### Primary Outcomes Analysis: Change in Daily Time Spent in Sedentary Behavior

Table 2 presents the means and standard deviations and effect sizes for: 1) percentages of daily time spent in sedentary behavior, and both light and moderate-to-vigorous intensity physical activity during the baseline period and each of the 3 physical activity break conditions (i.e. 3-min break after 30 continuous sedentary minutes, 6-min break after 60 continuous sedentary minutes, and 12-min break after 120 continuous sedentary minutes) and; 2) corresponding changes from baseline in daily minutes spent sedentary, and performing light- and moderate-to-vigorous physical activity for each of the 3 physical activity break conditions. Percent time spent in sedentary behavior was significantly decreased in all 3 physical activity break conditions relative to baseline (*p*<0.005). Planned pairwise comparisons revealed that the 3-min physical activity break condition produced significantly greater reductions in percent time spent sedentary compared to the 12-min physical activity break condition (*p* = 0.04).

**Table 2:** Time spent sedentary and active during baseline and the 3 physical activity break conditions.

Outcome	% of Daily Waking Hours	Baseline Minutes and Change in Daily Minutes from Baseline
**Sedentary**		
Baseline	72.2^a^ (68.5, 76.0)	593.7 (546.7, 640.6)
3-min PA break condition	66.3^b^ (61.7, 71.0)	−47.2 (−66.3, −28.2; 0.52)
6-min PA break condition	66.6^bc^ (61.5, 71.7)	−44.5 (−65.2, −23.8; 0.47)
12-min PA break condition	69.0^c^ (64.7, 73.2)	−26.2 (−40.7, −11.6; 0.31)
**Light PA**		
Baseline	22^a^ (19.8, 25.9)	183.6 (161.1, 206.1)
3-min PA break condition	26.7^b^ (23.0, 30.3)	+31.0 (15.8, 46.2; 0.43)
6-min PA break condition	26.7^bc^ (22.6, 30.9)	+31.0 (13.6, 48.4; 0.40)
12-min PA break condition	24.7^c^ (21.4, 27.9)	+15.3 (3.9, 26.8; 0.23)
**Moderate-to-vigorous PA**		
Baseline	5.0^a^ (3.6, 6.3)	41.6 (29.6, 53.6)
3-min PA break condition	7.0^b^ (5.4, 8.7)	+16.3 (8.5, 24.0; 0.49)
6-min PA break condition	6.7^b^ (5.0, 8.4)	+13.5 (6.3, 20.6; 0.42)
12-min PA break condition	6.4^b^ (4.7, 8.1)	+10.8 (4.2, 17.5; 0.31)

Note. Physical activity break conditions  = 3-min walking break after 30 continuous sedentary minutes; 6-min walking break after 60 continuous sedentary minutes, and 12-min walking break after 120 continuous sedentary minutes.

Values are presented as mean (95% CI) for % of daily waking hours and mean (95% CI; Cohen's d effect size) for baseline minutes and change in daily minutes from baseline.

For sedentary, light, and moderate-to-vigorous physical activity separately, values with different superscript letters indicate significant (*P*<0.05) differences between groups based on planned comparisons.

### Secondary Outcomes Analyses: Change in Light- and Moderate-to-Vigorous Physical Activity

Percent time spent in both light- (*p*<0.05) and moderate-to-vigorous (*p*<0.01) physical activity was significantly increased in all 3 physical activity break conditions compared to baseline. Planned pairwise comparisons indicated that the 3-min physical activity break condition produced significantly greater increases in percent time spent performing light physical activity, compared to the 12-min physical activity break condition (*p* = 0.04). No other pairwise differences reached statistical significance.

### Acceptability of B-MOBILE Intervention

On a scale of 1 to 5, anchored by “strongly disagree” and “strongly agree”, participants rated the degree to which the: 1) real-time *B-MOBILE* smartphone display and feedback increased their motivation to take physical activity breaks; and 2) time they spent in sedentary behavior decreased as a result of the *B-MOBILE* smartphone-delivered messages and feedback. Twenty-seven (90%) of the 30 participants endorsed either a 4 (n = 11) or 5 (n = 17) in response to question 1 indicating that the real-time smartphone display and feedback significantly increased their motivation to take physical activity breaks. Similarly, twenty-seven (90%) of the 30 participants endorsed either a 4 (n = 13) or 5 (n = 14) in response to question 2 indicating that the time they spent in sedentary behavior was significantly decreased as a result of the smartphone-delivered messages and feedback.

Participants were also asked to indicate which of the 3 physical activity break conditions they preferred the *most* and *least*. The majority (n = 17 or mean [95% CI] 56.7% [39.2%, 72.6%]) of participants indicated they *most* preferred the 6-min break condition, followed by the 3-min break condition (n = 10/33.3% [19.2%, 51.2%)), and the 12-min break condition (n = 3/10.0% [3.5%, 25.6%]). A nearly equivalent number of participants endorsed the 3-min break condition (n = 14/46.7% [30.2%, 63.9%]) and the 12-min break condition (n = 16/53.3% [36.1%, 70.0%) as the *least* preferred.

## Discussion

The current study, to our knowledge, is the first to test a smartphone-based intervention to reduce objectively-measured sedentary time in overweight/obese individuals by interrupting prolonged bouts of sedentary behavior with brief physical activity breaks. Given that the best strategy to maximize frequency and duration of breaks from sedentary behavior is unknown, an important feature of this study involved the comparison of 3 different conditions that varied both the frequency of physical activity break prompts and the minimum duration of physical activity breaks – i.e. 3-min break prompt after 30 continuous sedentary minutes, 6-min break prompt after 60 continuous sedentary minutes, and 12-min break prompt after 120 continuous sedentary minutes.

Overall, results showed that the *B-MOBILE* smartphone-based intervention produced significant reductions in sedentary time among overweight and obese adults, and that the reduced time spent in sedentary behavior was replaced by significant increases in both light- and moderate-to-vigorous intensity physical activity. Additionally, findings demonstrated differential efficacy of the 3 physical activity break conditions, with the 3-min break condition producing on average approximately twice the magnitude of both decrease in sedentary time [5.9% (47 min/d) vs. 3.3% (26 min/d) decrease] and increase in light physical activity [3.9% (31.0 min/d) vs. 1.9% (15.3 min/d) increase] compared to the 12-min break condition. These results suggest that interrupting sedentary behavior more frequently with shorter physical activity breaks is more effective in decreasing time spent in sedentary behavior and increasing time spent in light physical activity compared to interrupting sedentary behavior less frequently with longer physical activity breaks. It will be important for future studies to determine the impact of more frequent/shorter physical activity break schedules on time spent in sedentary behavior and physical activity over periods of a longer duration than the 7-day intervals that were tested in the present study.

The nearly 6% reduction in sedentary time produced by this low-intensity smartphone-based intervention, particularly the 3-min physical activity break condition, is larger than the 3-4% reductions in sedentary time reported in previous studies involving other low-intensity strategies [16]–[18]. The effectiveness of the smartphone-based intervention may be partially explained by several inherent unique advantages and features, including its ability to: 1) target *all* forms of sedentary behavior across *all* environmental settings; 2) automatically monitor sedentary behavior via the onboard accelerometer, thereby eliminating the burden of self-monitoring; 3) use monitored data to remind participants when to take physical activity breaks thus reducing the amount of required forethought or effort [21]; and 4) provide reinforcing feedback in real time to motivate habitual interruption of sedentary behavior with brief physical activity breaks. Given these features and the fact that smartphone technology is now owned by the majority (61%) of the population [23], future studies are needed to determine whether interventions like that implemented in the current study can promote changes in sedentary behavior on a larger scale.

Overall, subjects in the current study perceived that the *B-MOBILE* intervention, specifically the smartphone display and feedback, was very effective in increasing motivation to take physical activity breaks and decreasing time spent sedentary. Results also revealed an interesting discrepancy with half of the subjects indicating that they least preferred the 3-min physical activity break condition despite it being highly effective in decreasing sedentary behavior and increasing physical activity. However, while it appears that subjects may have been resistant to the idea of taking frequent breaks, this study shows that they are still able to accomplish the goal when prompted in real-time. Thus, what is effective in changing behavior may not necessarily be the same as what is preferred, particularly in the case of sedentary behavior which is highly habitual and often perceived as relaxing and pleasurable.

This study has several strengths. It is the first to show that a low-intensity smartphone-based intervention can significantly reduce sedentary behavior in overweight/obese individuals, a population at high risk for excessive sedentary time [3]–[4], [34]. This investigation is also one of the first to compare the impact of different physical activity break schedules on sedentary time, thereby providing important information for optimizing physical activity break frequency and duration in future studies. Key methodological strengths of this study include: 1) objective measurement of sedentary behavior and physical activity outcomes; 2) a study design that allowed subjects to serve as their own controls; 3) counterbalanced presentation of physical activity break conditions to control for order effects; and 4) minorities comprising a substantial proportion (33%) of the study sample.

This study also has certain limitations. Although the SWA has been shown to accurately estimate energy expenditure and provides estimates of sedentary time comparable to other objective monitors at the group level [30]–[33], it is not known whether the SWA can differentiate sitting and lying from standing. Consequently, the SWA may have misclassified standing as a sedentary behavior contributing to overestimation of sedentary time. The SWA also does not provide information on the amount of time allocated to specific forms of sedentary behavior, and thus it cannot be determined which sedentary behaviors were most effectively targeted by the intervention. This study did not include a long-term follow-up period or measure important disease risk variables that have been linked to excessive sedentary time [5]–[7]. Therefore, future studies are needed to determine whether this intervention has a sustainable effect on sedentary behavior and can improve health outcomes in a randomized controlled trial. Finally, while the focus of the current study was to test whether the smartphone-based intervention could significantly reduce total sedentary time, it will also be important for future studies to study *where*, *when* and for *whom* these types of automated electronic interventions are most successful.

## Conclusions

This smartphone-based intervention produced significant reductions in the amount of time that overweight/obese individuals spent sedentary. The reductions in sedentary time were replaced by significant increases in light- and moderate-to-vigorous intensity physical activity. This study provides important information to guide development of future sedentary-focused interventions, showing that encouraging individuals to interrupt sedentary behavior more frequently with shorter physical activity breaks may be more effective than interrupting sedentary behavior less frequently with longer physical activity breaks. Future investigations will focus on determining whether this intervention has a sustainable impact on sedentary behavior and related health outcomes.

## Supporting Information

Checklist S1 TREND Statement Checklist.
(PDF)

Protocol S1 IRB-Approved Research Protocol.
(DOCX)

File S1 Supplementary Data.
(SAV)

## References

[1] Sedentary Behavior Research Network (2012) Letter to the editor: standardized use of the terms “sedentary” and “sedentary behaviours.”. Appl Physiol Nutr Metab 37: 540–542. 22540258 10.1139/h2012-024

[2] Newton RL Jr, Han H, Zderic T, Hamilton M (2013) The energy expenditure of sedentary behavior: a whole room calorimeter study. PLOS ONE 8: e63171 10.1371/journal.pone.0063171 23658805 PMC3643905

[3] Chau JY, van der Ploeg HP, Merom D, Chey T, Bauman AE (2012) Cross-sectional associations between occupational and leisure-time sitting, physical activity, and obesity in working adults. Prev Med 54: 195–200. 22227284 10.1016/j.ypmed.2011.12.020

[4] Du H, Bennett D, Li L, Whitlock G, Guo Y, et al (2013) Physical activity and sedentary leisure time and their associations with BMI, waist circumference, and percentage body fat in 0.5 million adults: The China Kadoorie Biobank Study. Am J Clin Nutr 97: 487–496. 23364014 10.3945/ajcn.112.046854 PMC4345799

[5] Bankoski A, Harris TB, McClain JJ, Brychta RJ, Caserotti P, et al (2011) Sedentary activity associated with metabolic syndrome independent of physical activity. Diabetes Care 34: 497–503. 21270206 10.2337/dc10-0987 PMC3024375

[6] Healy GN, Matthews CE, Dunstan DW, Winkler EA, Owen N (2011) Sedentary time and cardiometabolic biomarkers in US adults: NHANES 2003-06. Eur Heart J 32: 590–597. 21224291 10.1093/eurheartj/ehq451 PMC3634159

[7] Henson J, Yates T, Biddle SJ, Edwardson CL, Khunti K, et al (2013) Associations of objectively-measured sedentary behaviour and physical activity with markers of cardiometabolic health. Diabetologia 56: 1012–1020. 23456209 10.1007/s00125-013-2845-9

[8] León-Muñoz LM, Martínez-Gómez D, Balboa-Castillo T, López-García E, Gullar-Castillón P, et al (2013) Continued sedentariness, change in sitting time, and mortality in older adults. Med Sci Sports Exerc 45: 1501–1507. 23439420 10.1249/MSS.0b013e3182897e87

[9] Matthews CE, George SM, Moore SC, Bowles HR, Blair A, et al (2012) Amount of time spent in sedentary behaviors and cause-specific mortality in US adults. Am J Clin Nutr 95: 437–445. 22218159 10.3945/ajcn.111.019620 PMC3260070

[10] Healy GN, Dunstan D, Salmon J, Cerin E, Shaw J, et al (2008) Breaks in sedentary time: beneficial associations with metabolic risk. Diabetes Care 31: 661–666. 18252901 10.2337/dc07-2046

[11] Dunstan DW, Kingwell BA, Larsen R, Healy GN, Cerin E, et al (2012) Breaking up prolonged sitting reduces postprandial glucose and insulin responses. Diabetes Care 35: 976–983. 22374636 10.2337/dc11-1931 PMC3329818

[12] Peddie MC, Bone JL, Rehrer NJ, Skeaff CM, Gray AR, et al (2013) Breaking prolonged sitting reduces postprandial glycemia in healthy normal-weight adults: a randomized crossover trial. Am J Clin Nutr 98: 358–366. 23803893 10.3945/ajcn.112.051763

[13] Van Dijk JW, Venema M, van Mechelen W, Stehouwer CD, Hartgens F, et al (2013) Effect of moderate-intensity exercise versus activities of daily living on 24-hour blood glucose homeostasis in male patients with type 2 diabetes. Diabetes Care 36: 3448–3453. 24041682 10.2337/dc12-2620 PMC3816888

[14] Swartz AM, Squires L, Strath SJ (2011) Energy expenditure of interruptions to sedentary behavior. Int J Behav Nutr Phys Act 8: 69. 21708007 10.1186/1479-5868-8-69 PMC3141617

[15] Latouche C, Jowett JB, Carey AL, Bertovic DA, Dunstan DW, et al (2013) Effects of breaking up prolonged sitting on skeletal muscle gene expression. J Appl Physiol 114: 453–460. 23271697 10.1152/japplphysiol.00978.2012

[16] Otten JJ, Jones KE, Littenberg B, Harvey-Berino J (2009) Effects of television viewing reduction on energy intake and expenditure in overweight and obese adults: a randomized controlled trial. Arch Intern Med 169: 2109–2115. 20008695 10.1001/archinternmed.2009.430

[17] Carr LJ, Karvinen K, Peavler M, Smith R, Cangelosi K (2013) Multicomponent intervention to reduce daily sedentary time: a randomised controlled trial. BMJ Open 3: e003261 10.1136/bmjopen-2013-003261 PMC3808782 24141969

[18] Gardiner PA, Eakin EG, Healy GN, Owen N (2011) Feasibility of reducing older adults' sedentary time. Am J Prev Med 41: 174–177. 21767725 10.1016/j.amepre.2011.03.020

[19] Healy GN, Eaking EG, Lamontagne AD, Owen N, Winkler EA, et al (2013) Reducing sitting time in office workers: short-term efficacy of a multicomponent intervention. Prev Med 57: 43–48. 23597658 10.1016/j.ypmed.2013.04.004

[20] Kozey-Keadle S, Staudenmayer J, Libertine A, Mavilia M, Lyden K, et al. (2013) Changes in sedentary time and physical activity in response to an exercise training and/or lifestyle intervention. J Phys Act Health [Epub ahead of print]. 10.1123/jpah.2012-0340 24184493

[21] Rutten GM, Savelberg HH, Biddle SJ, Kremers SP (2013) Interrupting long periods of sitting: good STUFF. Int J Behav Nutr Phys Act 10: 1 10.1186/1479-5868-10-1 23281722 PMC3542098

[22] Global System for Mobile Communications Association (2013) “The Mobile Economy 2013.” Global System for Mobile Communications Association, London, UK. Available: http://www.gsmamobileeconomy.com/GSMA Mobile Economy 2013.pdf. Accessed 7 February 2014.

[23] Smith A (2013) “A Smartphone Ownership – 2013 Update.” Pew Research Center, Washington D.C. Available: http://pewinternet.org/Reports/2013/Smartphone-Onwership-2013.aspx. Accessed 7 February 2014.

[24] Consolvo S, Landay JA, McDonald DW (2009) Designing for behavior change in everyday life. IEEE Computer 42: 86–89. PMC2815473 20351876

[25] Burke LE, Styn MA, Sereika SM, Conroy MB, Ye L, et al (2012) Using mHealth technology to enhance self-monitoring for weight loss: a randomized trial. Am J Prev Med 43(1): 20–26. 22704741 10.1016/j.amepre.2012.03.016 PMC3563396

[26] Greaves CJ, Sheppard KE, Abraham C, Hardeman W, Roden M, et al (2011) Systematic review of intervention components associated with increased effectiveness in dietary and physical activity interventions. BMC Public Health 11: 119 10.1186/1471-2458-11-119 21333011 PMC3048531

[27] Fujiki Y (2010) iPhone as a physical activity measurement platform. In: Proceedings of the ACM Conference on Human Factors in Computing Systems 10–15 April 2010. Atlanta: ACM Press. pp. 4315–4320.

[28] Consolvo S, McDonald DW, Toscos T, Chen MY, Froehlic J, et al. (2008) Activity sensing in the wild: a field trial of ubifit garden. In: Proceedings of the SIGCHI Conference on Human Factors in Computing Systems 10–15 April 2008 Florence: ACM Press. pp. 1797–1806.

[29] Klasnja P, Consolvo S, McDonald DW, Landay JA, Pratt W (2009) Using mobile and personal sensing technologies to support health behavior change in everyday life: lessons learned. In: Proceedings of the American Medical Informatics Association Annual Symposium 14–18 Nov 2009. San Francisco: American Informatics Association. pp. 338–342. PMC2815473 20351876

[30] Jakicic JM, Marcus M, Gallagher KI, Randall C, Thomas E, et al (2004) Evaluation of the SenseWear Pro Armband to assess energy expenditure during exercise. Med Sci Sports Exerc 36: 897–904. 15126727 10.1249/01.mss.0000126805.32659.43

[31] Johannsen DL, Calabro MA, Stewart J, Franke W, Rood JC, et al (2010) Accuracy of armband monitors for measuring daily energy expenditure in healthy adults. Med Sci Sports Exerc 42: 2134–2140. 20386334 10.1249/MSS.0b013e3181e0b3ff

[32] Unick JL, Bond DS, Jakicic JM, Vithiananthan S, Ryder BA, et al (2012) Comparison of two objective monitors for assessing physical activity and sedentary behaviors in bariatric surgery patients. Obes Surg 22: 347–352. 21814865 10.1007/s11695-011-0491-1 PMC3242159

[33] Wetten AA, Batterham M, Tan SY, Tapsell L (2014) Relative validity of three accelerometer models for estimating energy expenditure during light activity. J Phys Act Health 11: 638–647. 23417054 10.1123/jpah.2011-0167

[34] Bond DS, Thomas JG, Unick JL, Raynor HA, Vithiananthan S, et al (2013) Self-reported and objectively measured sedentary behavior in bariatric surgery candidates. Surg Obes Relat Dis 9: 123–128. 23265767 10.1016/j.soard.2012.09.008 PMC3558551

[35] Scheers T, Philippaerts R, Lefevre J (2013) SenseWear-determined physical activity and sedentary behavior and metabolic syndrome. Med Sci Sports Exerc 45: 481–489. 23034646 10.1249/MSS.0b013e31827563ba

