# Supplementary material for: B-MOBILE - A Smartphone-Based Intervention to Reduce Sedentary Time in Overweight/Obese Individuals: A Within-Subjects Experimental Trial
Source: PLoS One. 2014 Jun 25;9(6):e100821. doi: 10.1371/journal.pone.0100821 (PMC4071034; doi:10.1371/journal.pone.0100821)
Supplement: Protocol S1 — IRB-Approved Research Protocol. (DOCX) [file pone.0100821.s002.docx]

**A Mobile Health Approach to Reducing Sedentary Time in Obese Adults**

**SPECIFIC AIMS/BACKGROUND AND SIGNIFICANCE**

Sedentary behavior (SB) poses a distinct and significant health risk. There is growing consensus that SB, activities performed while sitting that require minimal energy expenditure (e.g., watching TV, using a computer, driving a car), have deleterious health consequences.^1-2^ Recent epidemiologic studies show that greater sedentary time—independent of physical activity (PA) levels—is associated with increased risk of type 2 diabetes^3^, cardiovascular disease^3-4^, metabolic syndrome^5^, weight gain^6-8^, and obesity.^9-10^ Moreover, there is strong evidence that SB is an independent risk factor for all-cause and cardiovascular-related mortality.^3, 11-13^

Obese individuals are at particular risk for engaging in high levels of SB. Increasing degrees of overweight/obesity have been associated with greater sedentary time and longer periods of sitting without interruption.^14-16^ Recent data from our group suggest that this may especially be the case with severely obese bariatric surgery patients who spend 80% of their objectively-assessed waking hours in SB^17-18^, considerably higher than the percentage of time sedentary (57%-69%) reported in the general adult population.^14-15, 19-20^

However, few studies have attempted to intervene on obese individuals’ high levels of SB. We recently conducted a small pilot study to reduce SB in severely obese individuals undergoing bariatric surgery. Participants received 6 wk of individual instruction in strategies (self-monitoring, goal-setting, etc.) to reduce sedentary time and accelerometer-derived feedback on SB at the end of this period. However, this approach did not change sedentary time and participants reported difficulty in identifying and monitoring their SB. Additionally, the efficacy of this approach may have been limited by the lack of immediate feedback on SB. Similarly, recent interventions to reduce SB via an electronic TV lock-out system^21^ or a tailored mailing^22^ produced only modest reductions in daily sedentary time (average reduction of 3.5%). Thus, novel approaches to reducing SB are clearly needed.

**The goal of the current study is to evaluate a novel mobile health (mHealth) approach to reduce SB in obese individuals. The proposed mHealth intervention addresses the limitations of previous studies by harnessing widely available smartphone technology to *automatically monitor* and *deliver immediate feedback* on SB as it occurs. Ownership of Internet-connected smartphones is expected to reach 50% this year.** Smartphones are ideal for intervention on SB because they allow for individuals’ SB to be monitored via the on-board accelerometer. This information can then be used to deliver individually tailored behavioral prompts and feedback in the natural environment to modify SB in real-time. It is also possible to present feedback in an entertaining and engaging format that encourages adherence to the intervention protocol.

In collaboration with a firm that develops smartphone applications for research, we recently conducted pilot testing that shows built-in smartphone accelerometers demonstrate very good agreement (96%) with a research-grade activity monitor (SenseWear Mini Armband [SWA]) in detecting SB, and that this information can be used to provide real-time feedback. We now propose to conduct a study to evaluate the feasibility and acceptability of this technology to reduce SB in obese individuals. We will compare three smartphone goal-driven prompting/feedback schedules to determine which schedule produces the best adherence and has the greatest impact on SB. The 3 schedules, each of which will be presented in counterbalanced order, are (1) a prompt after every 30 min of sitting to walk for 3 min, (2) a prompt after every 60 min of sitting to walk for 3 min, and (3) a prompt after every 120 min of sitting to walk for 12 min. Participants will follow each schedule for 7 days. Participants will wear the SWA to determine daily percentage of time spent in SB and complete a brief questionnaire to determine intervention acceptability/feasibility.

**Primary Aim:** To compare the 3 different smartphone prompting/feedback schedules on (a) adherence to prompts and (b) overall sedentary time over consecutive 7-day trials in obese individuals.

The proposed project addresses the public health problem of increasing time spent in SB by exploring the viability of popular mobile smartphone technology for decreasing SB in obese, sedentary individuals. If efficacious, a mHealth approach could be used to help large numbers of individuals monitor and limit their SB for very little cost using a device they already own.

**D.2. Participants.** A sample of 20 male and female obese individuals will participate in this study. Participants will be between the ages of 21 and 70. A portion of this sample will include obese individuals who have undergone bariatric surgery given our previous research showing that this patient population is at risk for engaging in high levels of sedentary behavior.

Participants will be excluded from participating if they:

1. Report being unable to engage in daily activities and walk continuously for ≥ 10 minutes without assistance.
2. Are currently involved in a physical activity intervention.
3. Body Mass Index < 25 kg/m^2^
4. Are within < 2 months of having undergone bariatric surgery
5. Are unable to read or understand the study materials.
6. Are currently taking certain medications (e.g., for hypertension) that cause dizziness and/or feeling faint when sitting or standing.
7. Report any condition that in the opinion of the investigators would preclude adherence to the intervention protocol, including plans to relocate, history of substance abuse or other significant psychiatric problems, or terminal illness.

Inclusion/exclusion criteria are designed to identify a heterogeneous sample of obese individuals and thus ensure that the findings are generalizable. We will include all obese individuals, regardless of PA level, given that SB is considered to be distinct from PA.^2^

**Study Design and Procedures.**

Study Design. The proposed study will evaluate the feasibility and acceptability of an mHealth approach involving use of smartphone technology to reduce SB in obese individuals. We will employ a within-subjects pre-post design to determine which of three goal-driven smartphone prompting/feedback schedules produces the best adherence and the greatest reduction in sedentary time. Twenty participants will report to the Weight Control and Diabetes Center (WCDRC) on 5 separate occasions over a 4-week period. The first will be an assessment visit during which participants will be given a SenseWear Mini Armband (SWA) to wear and monitor SB for a period of 7 consecutive days. After this control period, participants will return to the WCDRC to receive the smartphone. Over the next 3 weeks, participants will be presented with each of the 3 prompting/feedback schedules (see *Intervention* section below for description of prompting/feedback schedules) for 7 consecutive days in counterbalanced order. Participants will return to the center after each 7-day testing period to rate their acceptability of each prompting/feedback schedule, receive information on their adherence to wearing the SWA and carrying the smartphone, download data from the smartphone and SWA, and have the SWA recharged for continued use. Participants will receive a $20 honorarium at the conclusion of each of the five visits, for a total of $100.

Recruitment, Eligibility, Screening, and Orientation. We will recruit obese individuals from both the community and from bariatric surgery clinics and support groups affiliated with Lifespan hospitals. Study advertisements will be posted on the Lifespan internet/intranet and connected social media outlets (i.e. Facebook and Twitter). Individuals who are interested in the study and respond to the advertisements by calling the provided WCDRC number will receive a brief description of the study and be screened to determine initial eligibility. Patients who have undergone bariatric surgery at The Miriam and Rhode Island Hospitals will be recruited during regularly scheduled postoperative clinic visits. The attending surgeon or nurse practitioner will give patients a flyer that briefly describes the study. Patients who wish to be contacted further about the study will provide their signature and a contact number on the flyer that will be faxed to the WDCRC (this recruitment method has been used in previous and ongoing studies conducted at the WCDRC). A similar procedure will be followed in Lifespan hospital-affiliated bariatric surgery support groups, with the exception that one or members of the research team will briefly explain the study in person and collect signed flyers from interested individuals. Following faxed receipt of the flyer to research staff at the WCDRC, patients will be contacted by telephone and screened for eligibility.

Individuals who appear to meet study eligibility criteria will be invited to an orientation/assessment visit where informed consent will be obtained and the initial assessments (questionnaires and height/weight measurements) completed. During the orientation, the requirements of the study will be discussed in detail to determine if the participant is able to make the time commitment for this trial. After completing the orientation, participants will complete one week of SB monitoring via the SWA before beginning the intervention, as described below.

Intervention. The overall purpose of the intervention is to decrease time spent in SB among obese individuals, including those who have undergone bariatric surgery.. The intervention approach combines an advanced smartphone device with an onboard (i.e., built-in) accelerometer sensor and a sophisticated smartphone application (aka. “app”) designed in collaboration with behavioral scientists (Drs. Bond and Thomas) and engineers/computer scientists (MEI Research, Ltd). The components of the intervention include (a) SB Education and Goal Setting, (b) Real-time Monitoring of SB, (c) Intelligent Prompts to Limit SB, and (d) Real-Time Feedback on SB. These components are described in detail below:

- SB Education and Goal Setting: The purpose of this treatment component is to provide patients with a rationale for reducing SB, and to set goals for modifying SB (goal setting is known to facilitate behavior change).^32^ Upon completing the initial week of SB assessment via the SWA, participants will meet individually with a member of the study team to discuss the health consequences of SB and the health benefits that are associated with reducing SB. They will then set goals for reducing SB. In this study, we will test three different goals which vary the frequency and duration of prompted breaks in sedentary time. The three goals are listed in the table below. Participants will be randomly assigned to follow each of these goals for one week during the 3-week intervention period. Assignment of the goals will be counterbalanced.

**GOALS FOR STUDY 1**

|  | **Maximum Amount of SB Time Before Taking a Break** | **Recommended Duration of SB Breaks** |
| --- | --- | --- |
| **Goal #1** | **30 minutes** | **3 minutes** |
| **Goal #2** | **60 minutes** | **6 minutes** |
| **Goal #3** | **120 minutes** | **12 minutes** |

- Real-time Monitoring of SB: Participants will be instructed to carry an Android smartphone (their own or one provided to them by the study) with them at all times during the intervention period. The smartphone may be carried in a pocket or secured to clothing via a clip. The phone will automatically monitor SB behavior in real-time (in one-minute increments) via an onboard accelerometer. **Notably, the burden of monitoring SB rests completely on the smartphone and requires no effort on the part of the participant.** These data will be available to the research team in real-time (via the smartphones’ always-on Internet connection) and will be used to inform the following two intervention components:
- Intelligent Prompts to Limit SB: The SB goals assigned to participants will be used in conjunction with the real-time SB data obtained from the smartphone to prompt SB breaks when the maximum threshold of SB time has been reached. In Phase 1, the goal of one of the conditions involves taking a break of 3 minutes after every 30 minutes of SB. If the smartphone accelerometer detects that they have accrued 30 minutes of SB without taking a break of at least 3 minutes, the phone will produce an audible prompt with on-screen text reminding them of their goal, and providing encouragement for taking a SB break. Participants may then silence the prompt, or delay the prompt to reoccur after a set period. Participants will also have the option of changing the prompt from an audible tone to a vibration (e.g., during meetings or class).
- Real-Time Feedback on SB: Participants will receive two types of feedback. First, real-time smartphone-derived accelerometry data will be used to determine whether participants are compliant with the prompts described above. When a participant responds to a prompt by substituting SB with walking activity for the recommended duration they will receive a supportive message praising their accomplishment. The second type of feedback involves a persistent display on the smartphone screen that is continuously updated to reflect the length of the current bout of SB and the total number of both sedentary and non-sedentary minutes. A car dashboard metaphor will be used; a “gas gauge” will display the number of minutes remaining until the next SB break. The “tank” is “refilled” (i.e., the gauge is reset) when participants take a SB break. Likewise, two “odometers” will track the total number of non-sedentary minutes accumulated and the total SB minutes for the day. A bright green “go” light will appear on the dashboard each time participants fully comply with an SB prompt, up to a total of 10 “go” lights per day. Persistent displays such as these have been shown to motivate health behavior in other studies using similar technology.^33-34^

Assessment Components. The Phase 1 study will be conducted over a 4-week period: week 1 (control) and weeks 2 through 4 (intervention). During week 1, participants will complete questionnaires to assess demographic characteristics (including occupation) and amount of time spent in specific SB, undergo anthropometric measurements, and wear the SWA for 7 consecutive days. At the end of each intervention week, questionnaires will be administered to assess acceptability/feasibility of the smartphone prompting/feedback schedule completed that week and the amount of time spent in specific SB. Changes in sedentary time between the control week and each of the intervention weeks will be assessed via the SWA.

Sedentary time, breaks and behaviors (For Research Assessment Only; Not Involved in Intervention). The SenseWear Mini Armband (SWA, BodyMedia, Inc., Pittsburgh, PA) will be used to objectively measure sedentary time and breaks during waking hours. The SWA is a wireless multi-sensor monitor worn on the upper arm over the triceps muscle that simultaneously integrates motion data from a triaxial accelerometer and physiological metrics from skin temperature, galvanic skin response, and heat flux sensors to provide estimates of energy expenditure. Data is processed with software algorithms that match each recorded minute of data with an activity class (e.g., walking, running, rest, etc.). Each activity class has a linear regression model that enables mapping of values from motion and physiologic sensors to energy expenditure.^35^ The SWA “mini”, the newest version of the SWA, is smaller, lighter, and more accurate in measurement of low-intensity activities and total energy expenditure than its predecessor.^35^ Older versions of the SWA have been shown to accurately estimate energy expenditure when evaluated against indirect calorimetry and provide similar estimates of time spent in low-intensity activities and SB to other commonly used monitors.^18, 36-37^ Processed data will be summarized as the percentage of monitored waking time spent sedentary (≤ 1.5 METs).

Information on specific SB will be captured using a version of the Past-day Adults’ Sedentary Time (PAST) Questionnaire.^38^ This measure asks about the time spent sitting or lying (while awake) during work, travelling, watching television, using the computer (excluding work), reading (excluding work), hobbies, and any other activities not reported in the previous items on a typical weekday and a typical weekend day during the previous week. Participants will complete this measure at baseline and at the end of each of the 4 intervention weeks.

Acceptability/feasibility of smartphone goal-driven prompting/feedback schedules. A brief acceptability/feasibility survey will be used to obtain information on participants’ opinions and experiences with the smartphone and different goal-driven prompting/feedback schedules. Participants will indicate on a 5-point Likert scale (ranging from 1/strongly disagree to 5/strongly agree) the extent to which they agree with multiple statements (e.g., The smartphone application was easy to understand and use. The smartphone application made me feel more confident in my ability to sit less., etc.)

Health related quality of life. The medical outcomes study Short Form –36 Questionnaire (SF-36) will be used to assess participants’ health related quality of life at baseline and the final visit.

Anthropometric measures**.** Body weight will be measured to the nearest 0.1 kg using an electronic scale and height will be measured to the nearest 0.1 cm with a wall-mounted stadiometer. BMI will be calculated as weight divided by height squared (kg/m^2^).

Demographic characteristics. Information on participants’ age, sex, race/ethnicity, type of employment, educational level and marital status will be collected.

Statistical Analysis, Sample Size and Power Estimates.

General Analysis Considerations. Descriptive statistics will be used to characterize the sample and to identify out of range values and possible threats to test assumptions.

Primary Aim: The goal is to determine which of the 3 SB goal/prompting condition results in the greatest adherence to prompts (defined as the number of prompts resulting in an SB break / total number of prompts) and the largest reduction in % of time spent in SB (defined as % of time spent in SB during intervention week - % of time spent in SB during baseline week). A repeated measures analysis of variance (RM-ANOVA) controlling for the order of presentation of the 3 conditions will be used to compare the effect of condition on adherence and reduction in % time spent in SB to the 3 goal/prompting schedules. With 20 participants, we will be able to detect a statistically significant difference in adherence/reduction in % SB of at least 5% (i.e., participants responded to ≥ 5% more prompts or spent ≥ 5% less time in SB) if the SD for condition is ≤ 10% and the autocorrelation is ≥ 0.7.

**References Cited**

1. Pate RR, O'Neill JR, Lobelo F. The evolving definition of "sedentary". *Exerc Sport Sci Rev.* Oct 2008;36(4):173-178.

2. Owen N, Healy GN, Matthews CE, Dunstan DW. Too much sitting: the population health science of sedentary behavior. *Exerc Sport Sci Rev.* Jul 2010;38(3):105-113.

3. Grontved A, Hu FB. Television viewing and risk of type 2 diabetes, cardiovascular disease, and all-cause mortality: a meta-analysis. *JAMA.* Jun 15 2011;305(23):2448-2455.

4. Wijndaele K, Brage S, Besson H, et al. Television viewing and incident cardiovascular disease: prospective associations and mediation analysis in the EPIC Norfolk Study. *PLoS One.* 2011;6(5):e20058.

5. Bankoski A, Harris TB, McClain JJ, et al. Sedentary activity associated with metabolic syndrome independent of physical activity. *Diabetes Care.* Feb 2011;34(2):497-503.

6. Mekary RA, Feskanich D, Malspeis S, Hu FB, Willett WC, Field AE. Physical activity patterns and prevention of weight gain in premenopausal women. *Int J Obes (Lond).* Sep 2009;33(9):1039-1047.

7. Wijndaele K, Lynch BM, Owen N, Dunstan DW, Sharp S, Aitken JF. Television viewing time and weight gain in colorectal cancer survivors: a prospective population-based study. *Cancer Causes Control.* Oct 2009;20(8):1355-1362.

8. Mozaffarian D, Hao T, Rimm EB, Willett WC, Hu FB. Changes in diet and lifestyle and long-term weight gain in women and men. *N Engl J Med.* Jun 23 2011;364(25):2392-2404.

9. Hu FB, Li TY, Colditz GA, Willett WC, Manson JE. Television watching and other sedentary behaviors in relation to risk of obesity and type 2 diabetes mellitus in women. *JAMA.* Apr 9 2003;289(14):1785-1791.

10. Boone JE, Gordon-Larsen P, Adair LS, Popkin BM. Screen time and physical activity during adolescence: longitudinal effects on obesity in young adulthood. *Int J Behav Nutr Phys Act.* 2007;4:26.

11. Wijndaele K, Brage S, Besson H, et al. Television viewing time independently predicts all-cause and cardiovascular mortality: the EPIC Norfolk study. *Int J Epidemiol.* Feb 2011;40(1):150-159.

12. Thorp AA, Owen N, Neuhaus M, Dunstan DW. Sedentary behaviors and subsequent health outcomes in adults a systematic review of longitudinal studies, 1996-2011. *Am J Prev Med.* Aug 2011;41(2):207-215.

13. Veerman JL, Healy GN, Cobiac LJ, et al. Television viewing time and reduced life expectancy: a life table analysis. *Br J Sports Med.* Aug 15 2011.

14. Healy GN, Matthews CE, Dunstan DW, Winkler EA, Owen N. Sedentary time and cardio-metabolic biomarkers in US adults: NHANES 2003-06. *Eur Heart J.* Mar 2011;32(5):590-597.

15. Healy GN, Wijndaele K, Dunstan DW, et al. Objectively measured sedentary time, physical activity, and metabolic risk: the Australian Diabetes, Obesity and Lifestyle Study (AusDiab). *Diabetes Care.* Feb 2008;31(2):369-371.

16. Healy GN, Dunstan DW, Salmon J, et al. Breaks in sedentary time: beneficial associations with metabolic risk. *Diabetes Care.* Apr 2008;31(4):661-666.

17. Bond DS, Unick JL, Jakicic JM, et al. Objective assessment of time spent being sedentary in bariatric surgery candidates. *Obes Surg.* Jun 2011;21(6):811-814.

18. Unick JL, Bond DS, Jakicic JM, et al. Comparison of Two Objective Monitors for Assessing Physical Activity and Sedentary Behaviors in Bariatric Surgery Patients. *Obes Surg.* Aug 4 2011.

19. Matthews CE, Chen KY, Freedson PS, et al. Amount of time spent in sedentary behaviors in the United States, 2003-2004. *Am J Epidemiol.* Apr 1 2008;167(7):875-881.

20. Colley RC, Garriguet D, Janssen I, Craig CL, Clarke J, Tremblay MS. Physical activity of Canadian adults: accelerometer results from the 2007 to 2009 Canadian Health Measures Survey. *Health Rep.* Mar 2011;22(1):7-14.

21. Otten JJ, Jones KE, Littenberg B, Harvey-Berino J. Effects of television viewing reduction on energy intake and expenditure in overweight and obese adults: a randomized controlled trial. *Arch Intern Med.* Dec 14 2009;169(22):2109-2115.

22. Gardiner PA, Eakin EG, Healy GN, Owen N. Feasibility of reducing older adults' sedentary time. *Am J Prev Med.* Aug 2011;41(2):174-177.

23. Hamilton MT, Hamilton DG, Zderic TW. Role of low energy expenditure and sitting in obesity, metabolic syndrome, type 2 diabetes, and cardiovascular disease. *Diabetes.* Nov 2007;56(11):2655-2667.

24. Thyfault JP, Booth FW. Lack of regular physical exercise or too much inactivity. *Curr Opin Clin Nutr Metab Care.* Jul 2011;14(4):374-378.

25. Bey L, Hamilton MT. Suppression of skeletal muscle lipoprotein lipase activity during physical inactivity: a molecular reason to maintain daily low-intensity activity. *J Physiol.* Sep 1 2003;551(Pt 2):673-682.

26. Zderic TW, Hamilton MT. Physical inactivity amplifies the sensitivity of skeletal muscle to the lipid-induced downregulation of lipoprotein lipase activity. *J Appl Physiol.* Jan 2006;100(1):249-257.

27. Stephens BR, Granados K, Zderic TW, Hamilton MT, Braun B. Effects of 1 day of inactivity on insulin action in healthy men and women: interaction with energy intake. *Metabolism.* Jul 2011;60(7):941-949.

28. Bergouignan A, Rudwill F, Simon C, Blanc S. Physical inactivity as the culprit of metabolic inflexibility: Evidences from bed-rest studies. *J Appl Physiol.* Aug 11 2011.

29. Bond DS, Jakicic JM, Unick JL, et al. Pre- to Postoperative Physical Activity Changes in Bariatric Surgery Patients: Self Report vs. Objective Measures. *Obesity (Silver Spring).* Apr 8 2010.

30. Owen N, Sugiyama T, Eakin EE, Gardiner PA, Tremblay MS, Sallis JF. Adults' sedentary behavior determinants and interventions. *Am J Prev Med.* Aug 2011;41(2):189-196.

31. Thomas JG, Wing RR. Beyond text messaging: The development and early outcomes of a next-generation mobile phone intervention for obesity. *Obesity (Silver Spring).* 2010;2010(18):S86.

32. Greaves CJ, Sheppard KE, Abraham C, et al. Systematic review of reviews of intervention components associated with increased effectiveness in dietary and physical activity interventions. *BMC Public Health.* 2011;11:119.

33. Klasnja P, Consolvo S, McDonald DW, Landay JA, Pratt W. Using mobile and personal sensing technologies to support health behavior change in everyday life: lessons learned. *Annual Conference of the American Medical Informatics Association.* 2009:338-342.

34. Consolvo S, Landay JA, McDonald DW. Designing for behavior change in everyday life. *IEEE Computer.* 2009;42(6):86-89.

35. Johannsen DL, Calabro MA, Stewart J, Franke W, Rood JC, Welk GJ. Accuracy of armband monitors for measuring daily energy expenditure in healthy adults. *Med Sci Sports Exerc.* Nov 2010;42(11):2134-2140.

36. Jakicic JM, Marcus M, Gallagher KI, et al. Evaluation of the SenseWear Pro Armband to assess energy expenditure during exercise. *Med Sci Sports Exerc.* May 2004;36(5):897-904.

37. Welk GJ, McClain JJ, Eisenmann JC, Wickel EE. Field validation of the MTI Actigraph and BodyMedia armband monitor using the IDEEA monitor. *Obesity (Silver Spring).* Apr 2007;15(4):918-928.

38. Clark, BK, Winkler E, Healy GN, Gardiner PG, Dunstan DW, Owen N, Reeves, MM. Adults' past day recall of sedentary time: reliability, validity, and responsiveness. *Med Sci Sports Exerc* 2012 [Epub ahead of print].
